# Validation of a structured questionnaire for COPD and prevalence of COPD in rural area of Mysore: A pilot study

**DOI:** 10.4103/0970-2113.53226

**Published:** 2009

**Authors:** P. A. Mahesh, B. S. Jayaraj, S. T. Prahlad, S. K. Chaya, A. K. Prabhakar, A. N. Agarwal, S. K. Jindal

**Affiliations:** *Department of Pulmonary Medicine, JSS Medical College and Hospital, Mysore - 570004, Karnataka, India*; 1 *Department of Pulmonary Medicine, Post Graduate Institute of Medical Education and Research, Chandigarh - 160 017, India*

**Keywords:** Biomass fuels, chronic obstructive pulmonary disease, nicotine dependence, prevalence, screening questionnaire

## Abstract

**Background:**

The prevalence of chronic obstructive pulmonary disease (COPD) is increasing in India and there is a need to study the prevalence of COPD, particularly in the rural areas, which may be most affected due to their lifestyle.

**Materials and Methods:**

First stage: Validation of the questionnaire–105 consecutive patients underwent administration of the structured questionnaire and spirometry was used as a gold standard for the diagnosis of COPD. Second stage: Adults above 40 years (n = 900) in two villages of Mysore district were administered with the validated questionnaire, Knowledge and Attitude questionnaire and Fagerstorm questionnaire, to assess nicotine dependency.

**Results:**

The questionnaire was found to have a sensitivity of 62.5% and specificity of 87.6% to diagnose COPD. Of the total 900 adults surveyed (Males: 453, Females: 447), the total prevalence of COPD was 7.1%. Males had a higher prevalence (11.1%) compared to females (4.5%). The prevalence of smoking was very high among men at 71.9% and all the women were nonsmokers. The prevalence of COPD was 14.7% in smokers, 19.3% had mild to moderate nicotine dependency and 12.8% were highly dependent. Of the women exposed to regular biomass fuels, the prevalence of COPD was 3.9%, which increased to 4.8% on addition of regular passive smoking. In smoking, male gender and age were significantly associated with COPD (*P* < 0.05).

**Conclusion:**

The structured questionnaire is a useful tool for the screening of COPD in field studies. Smoking and biomass fuel exposure are important risk factors for COPD.

## INTRODUCTION

Chronic obstructive pulmonary disease (COPD) is an important cause of morbidity and mortality. It is listed as the fourth leading cause of death worldwide.[1] The estimates for 2020, predict an even further increase in the number of people suffering from the disease.[2] India is one of the countries identified to have a significant increase in the burden of tobacco related mortality.[2]

There is a paucity of data regarding the prevalence and socioeconomic burden of COPD available in India. Only few population-based surveys have been carried out in India so far. India is a heterogenous country and it is important that different regions are represented in prevalence studies. In one of the pioneering studies in India, a large multicentric general population based survey[3] was undertaken using a structured questionnaire in adults (aged more than 35 years) and discovered that the prevalence of COPD was 4.1%. A total of 35,295 subjects in Bangalore, Chandigarh, Delhi, and Kanpur, were studied using uniform methodology and a standardized questionnaire. A prevalence of 5% in males and 3.2% in females was observed.

Prior to this study, there were only few single center studies,[4–13] most of which were conducted in North India. Only two studies[7,13] were conducted in Tamilnadu, South India. Prevalence rates varied from 2-22% in males and 1.2-19% in females. In 1964, Wig *et al.,*[4] compared the prevalence of rural and urban chronic bronchitis, smoking, and related factors. The prevalence rates in urban and rural areas were similar, approximately 10%. The prevalence rates for people above 55 years were 17% (for males) and 12% (for females), with a male preponderance. Bhattacharya[6] studied chronic bronchitis in rural population (aged above 30 years) and found the prevalence of chronic bronchitis to be 57/1000, with male preponderance. Overall prevalence is greater in males due to higher prevalence of smoking. Malik[11] found that bidi smokers had decreased lung functions and 13.55% of them had chronic bronchitis. In a community-based study, Jindal[12] found prevalence of COPD to be 5% in males and 2.8% in females. It was similar in both sexes, depending upon who were nonsmokers. Ray *et al.*,[13] found age specific prevalence of 33.0/1000 (40.8/1000 in males and 22.5% in females) in people above 30 years; all female were nonsmokers. Behera *et al.*,[14] studied effects of various fuels in rural homes of Chandigarh and observed the prevalence of COPD as 11.9%.

The present study was conducted as a pilot study for the validation of a structured questionnaire for the diagnosis of COPD in our population to estimate the prevalence of COPD in adults in rural Mysore and to identify the importance of risk factors associated with COPD and assess the knowledge and attitudes regarding smoking and nicotine dependence among smokers.

## MATERIALS AND METHODS

The study was carried out in two stages. In the first stage, a structured questionnaire that would be used in the field was validated and its diagnostic utility was determined. In the second stage, all the adults in two villages of Mysore district were administered the questionnaire as a pilot study to estimate the prevalence of COPD. A knowledge, attitude questionnaire and Fagerstorm questionnaire were also administered in the same population.

The structured questionnaire developed by Dr. Jindal for field studies which was validated in earlier studies, was utilized. The questionnaire elicited information on the demographic data and various respiratory symptoms; and a detailed analysis of the most important risk factors for COPD, tobacco smoking, passive smoking and exposure to biomass fuels, relevant to the rural population.

The validation in the present study was carried out on 105 consecutive adult patients, able to perform spirometry according to the American thoracic society (ATS),[15] criteria and were administered this questionnaire. The questionnaire was translated into the local language according to standard procedures for translation and back-translation. The respiratory nurse was trained to administer the questionnaire. The questionnaire was read out to the patient in exactly the same order as listed and sufficient time was given to the patient to respond to the questions. If the patient did not understand the questions, it was repeated; and if he was still doubtful, it was recorded as “No”. The respiratory nurse was unaware of the spirometry results while administering the questionnaire. The patients who underwent spirometry included patients with COPD, asthma, interstitial lung disease, bronchiectasis, and posttubercular sequelae, allergic rhinitis, evaluation of cough, dyspnea, preoperative evaluation or were normal. Spirometry was performed according to the ATS criteria.[15] After spirometry, the COPD patients were graded according to the Global Initiative for Chronic Obstructive Lung Disease (GOLD)[16] criteria. The diagnostic utility of the questionnaire was assessed by calculating sensitivity, specificity, positive predictive value, negative predictive value, and accuracy. The COPD diagnosed according to the GOLD[16] criteria was taken as the gold standard for the above calculations.

The validated questionnaire was used for screening the COPD cases in the second stage prevalence study. In addition, a questionnaire for assessing the knowledge regarding the adverse health effects of tobacco smoking and attitude about smoking, and Fagerstorm questionnaire to assess nicotine dependence, were administered to all the smokers. The study was conducted in two villages, Hadinaru and Suttur, near Mysore city. All the adults above 40 years were included in the study. The survey was conducted in the morning and evening to ensure compliance. The household list was obtained from the gramsabha register. The houses were visited at least on three occasions before declaring them as nonresponders. The questionnaire was administered by the coauthor, in the same manner as described in the first stage.

### Data analysis

The definition of COPD according to the structured questionnaire in the first stage was based on presence of all the following factors–(i) age above 40 years, (ii) smoking status of above 10 pack years or exposure to biomass fuels, (iii) presence of whistling in the chest or breathlessness, or early morning cough for at least three months in an year, for at least two consecutive years.

The definition of COPD according to the GOLD guidelines[17] was based on spirometry. Stage I-FEV1 >80% with FEV1/FVC <70%; stage II-FEV1 50–80% predicted, FEV1/FVC <70% with or without symptoms; stage III-FEV1 30–49%, FEV1/FVC <70% with or without symptoms; and stage IV-FEV1 <30% predicted, FEV1/FVC <70% or FEV1 <50% with chronic respiratory failure.

Other diseases were diagnosed with appropriate investigations. Asthma was diagnosed according to GINA[18] criteria; ILD and bronchiectasis were confirmed with high resolution CT scan; and posttubercular sequelae were confirmed with past history of tuberculosis, negative sputum smear for AFB and radiological evidence.

True positives were those “COPD cases” identified by both, the spirometry as well as the questionnaire. True negatives were those classified as “not a COPD case” by both, the spirometry as well as the questionnaire. False positives were those identified by only the questionnaire, but not by spirometry; and false negatives were those identified by spirometry, but not by the questionnaire. Sensitivity, specificity, positive predictive value (PPV), negative predictive value (NPV), and accuracy were calculated according to the standard methods.

In the second stage, prevalence of COPD was calculated as the number of subjects categorized as having COPD divided by the total number of subjects surveyed. Potential risk factors for COPD such as smoking, exposure to biomass fuels, and passive smoking were categorized based on the information available from the questionnaire. The percentage of subjects correctly answering the knowledge questions and their attitudes towards smoking were noted. A Fagerstorm questionnaire[19] score of 6 or less indicated mild to moderate nicotine dependence and above 6 indicated severe nicotine dependence.

## RESULTS

In the first stage, 105 consecutive patients underwent spirometry and were administered the structured questionnaire by a trained respiratory nurse. Sixteen of these subjects were suffering from COPD. The remaining subjects were normal,[11] suffering from allergic rhinitis and postnasal drip[20] and underwent spirometry for evaluation of cough, had asthma, restrictive lung diseases[5] including interstitial lung disease (pulmonary) or ascites (extra pulmonary), bronchiectasis and posttubercular sequelae,[2] underwent spirometry as a preoperative evaluation[13] or had spirometry as an evaluation of sensation of breathlessness due to anxiety.[3] Spirometry was used for the confirmation of COPD according to the GOLD criteria. Out of 16 patients, 10 belonged to GOLD stage II, 4 to stage III and 2 to stage IV. The mean age of the subjects studied was 43.09 years (SD 17.47). There were 62 males and 43 females.

**Figure 1:**
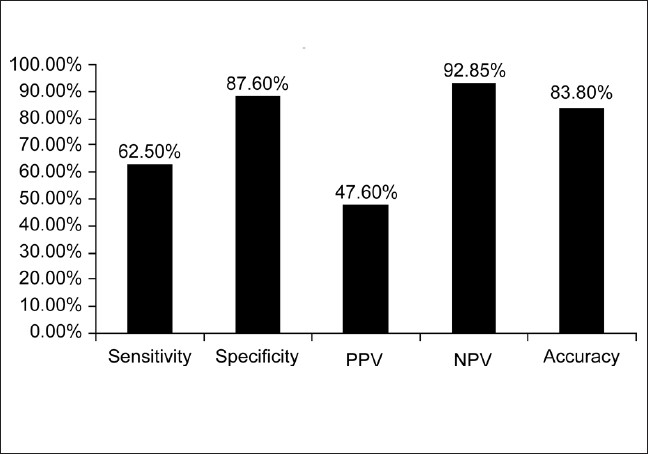
Diagnostic utility of the COPD screening questionnaire

The structured questionnaire could identify 10 out of 16 cases of COPD identified by spirometry giving a sensitivity of 62.5%. The specificity was 87.6%, PPV was 47.6%, NPV was 92.85%, and overall accuracy was 83.8% [Figure 1]. The 10 cases which were correctly diagnosed as COPD, were all males above 55 years and smokers. Of the 6 cases of COPD missed, 4 were males (one smoker) and 2 were females. A total of 11 cases (males, smokers, and above 48 years), were wrongly categorized as COPD. These involved, 7 asthma, 1 posttubercular sequelae, and 3 ILD cases. Seventy-eight cases were correctly classified as not having COPD. The sensitivity of the questionnaire increased with increasing severity of COPD as assessed by the GOLD criteria. It could correctly identify 5 of 10 COPD cases (50%) in stage II; 3 of 4 COPD cases (75%) in stage III; and both the cases (100%) in stage IV.

**Figure 2:**
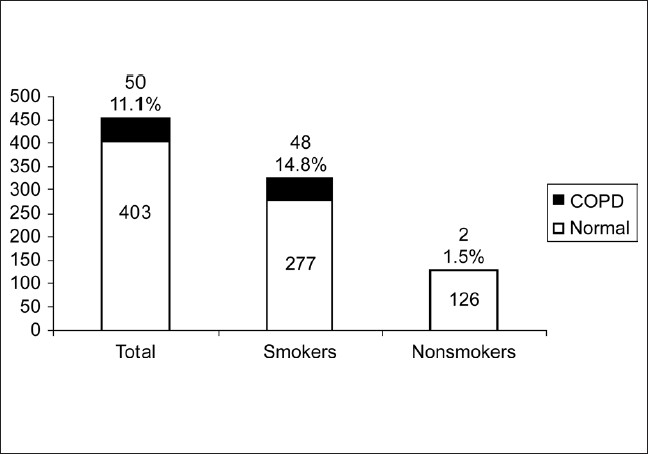
Prevalence of COPD in males, smokers and nonsmokers

**Figure 3:**
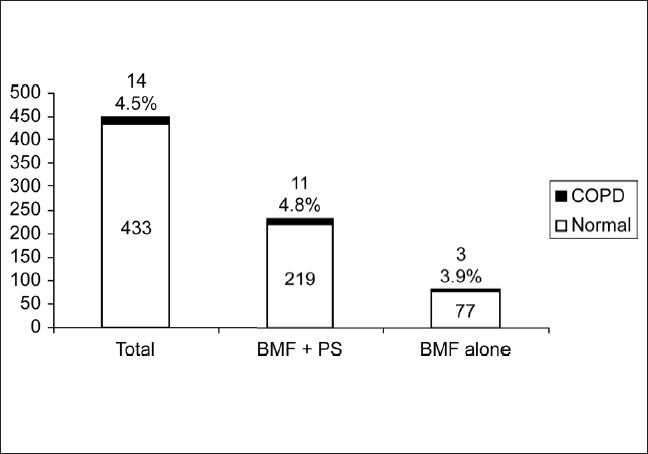
Prevalence of COPD in females, exposed to biomass fuels and passive smoking and biomass fuels alone without exposure to passive smoking. (BMF–biomass fuels, PS–passive smoking)

**Figure 4:**
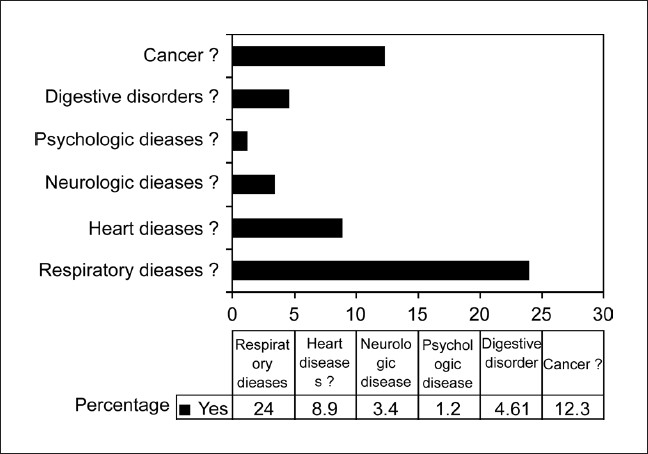
Knowledge among smokers regarding adverse effects of tobacco smoking

**Figure 5:**
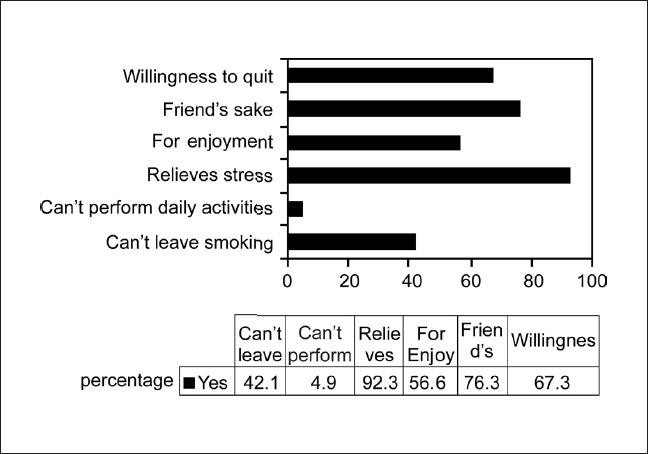
Attitudes regarding smoking among smokers in rural population

**Figure 6:**
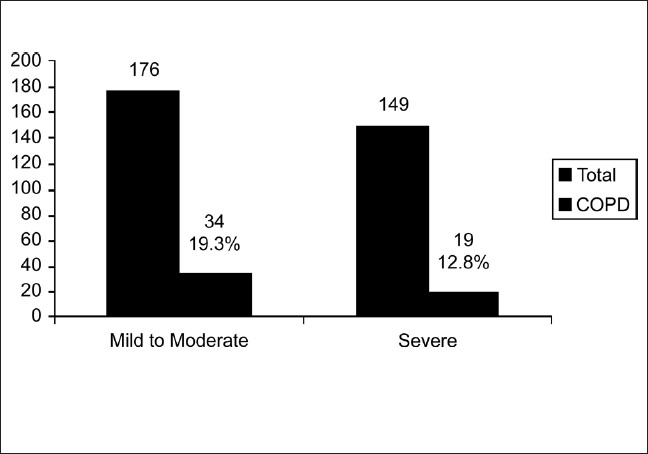
Severity of nicotine dependence according to Fagerstorm questionnaire

In the second stage, all the adults above 40 years in two villages, were administered the questionnaire by one of the coauthors, a total of 900 adults were interviewed with 453 (50.3%) males and 447 (49.7%) females. The response rate was 99.5%. The demographic characteristics including age, occupation and smoking status of male [Table 1] and female [Table 2] subjects in the study are presented. The proportion of subjects who ever smoked was 71.9%. Most of the men had smoked more than 20 pack years (61.5%). The mean pack years in subjects smoking more than 20 pack years were 48.02 (SD 25.73), and in those smoking less than 20 pack years were 11.14 (SD 5.36). Beedis (96.9%) were the most co3mmon tobacco used. It was observed in males that prevalence rate of COPD increase with increasing age and smoking (*P* < 0.05). The prevalence of COPD in males aged 40-49 years was 5.68% and increased to 28.57% in those aged more than 70 years. The prevalence of COPD in smokers who had smoked less than 20 pack years was 9.6%, which increased to 18% in subjects who smoked more than 20 pack years. The predominant occupation in males was agriculture followed by manual labor. Very few COPD cases were noted in nonsmokers. Most of the women in the study group were housewives (81.4%), and a small number of women worked as unskilled laborers (8.7%) and in agriculture (6.48%). Firewood was the most common domestic fuel used (91.95%) and most women had an exposure of more than 20 years to biomass fuels (80.9%). There were no female smokers in the study population. The proportion of females exposed to passive smoking at home was 55%. The common respiratory symptoms in males (smokers and nonsmokers) and females elicited by the questionnaire are given in [Table 3]. Cough and Sputum were the most common symptoms noted in both males and females followed by breathlessness and wheezing. The overall prevalence of COPD in the population interviewed was 7.1% of 900 subjects. It was 11.1% among men [Figure 2], 4.5% among women [Figure 3]. The prevalence was found to increase with advancing age. In males, highest prevalence was seen in subjects aged 70 and above (28.7%). The highest number of COPD cases in males was noted in the unemployed/retired group, which was due to the fact that many subjects in this group were elderly. The prevalence among male smokers was 14.7%. In females a similar trend was observed. Highest prevalence in females (above 70 years) was noted at 7.35%. Among females, 91.95% were exposed to biomass fuels. Prevalence of COPD among those who used biomass fuels without exposure to passive smoking was 3.9% and in those exposed to both biomass fuels and passive smoking was 4.8%. People using other fuels including kerosene were less in number and therefore no conclusions could be drawn. On assessing the knowledge and attitudes of smokers in the rural population regarding the health effects of smoking [Figure 4] it was observed that most of the people were unaware of the ill effects associated with smoking. Approximately 24% smokers were aware that smoking caused respiratory diseases and 12.3% were aware that smoking is associated with cancers. The attitudes of the subjects towards smoking are summarized [Figure 5]. Most of the smokers smoked to relieve stress (92.3%) and to keep company (76.3%). On educating about the adverse effects of smoking, the number of patients willing to quit was 67.3%. On applying Fagerstorm questionnaire for nicotine dependence, it was noted that 177 smokers (54.5%) had mild to moderate nicotine dependence and 148 (45.5%) had severe nicotine dependence [Figure 6]. The number of COPD cases in mild to moderate nicotine dependent subjects was 18 (10.16%) and in severe nicotine dependent subjects it was 30 (20.3%). The mean Fagerstorm score among patients with COPD was 6.29 (SD 2.76) and in patients without COPD it was 5.20 (SD 2.73). Presence of COPD was significantly related to severity of nicotine dependence (*P <* 0.005).

**Table 1:** Prevalence of COPD according to age, occupation, passive smoking (both at home and working place), smoking habits (males)

Variable	Classification	Prevalence			*P* value
		
		Number	No. of cases	%	
Age	40-49	176	10	5.68	>0.05
	50-59	119	15	12.6	
	60-69	102	15	14.7	
	70+	56	16	28.57	
Occupation	Unemployed/retired	11	4	36.36	>0.05
	Housewife (Females)	0	0	0	
	Unskilled worker	79	12	15.19	
	Skilled worker	44	8	18.18	
	Bussiness	28	3	10.71	
	Farmer	267	27	10.11	
	Government/private worker	23	2	6.25	
	Government/private supervisor	1	0	0	
	Government/private officer	0	0	0	
Smoking	Yes	325	53	16.31	<0.05
	No	128	3	2.34	
Passive smoking	Yes	107	14	13.08	<0.05
(At home)	No	346	42	12.14	
Passive smoking	Yes	287	43	14.18	<0.05
(At work)	No	166	13	7.83	
Types of smoking	Beedi	315	50	15.87	<0.05
	cigarette	9	2	22.22	
	Others	2	1	50	
	None	127	3	2.36	

**Table 3:** Prevalence and gender distribution of symptoms in smokers and nonsmokers

	Male				Female	
		
	Smokers (325)	%	Non smokers (128)	%	Non smokers (447)	%
Cough						
Presence	48	14.76	2	1.56	20	4.47
>3 months/yr	48	14.76	2	1.56	20	4.47
Sputum						
Presence	48	14.76	2	1.56	20	4.47
>3 months/yr	47	14.46	1	0.78	20	4.47
Breathlessness						
Presence	40	12.3	2	1.56	18	4.02
Severity						
Ground	10	3.07	1	0.78	2	0.44
Stair	30	9.23	0	0	16	3.58
At rest	6	1.84	1	0.78	0	0
None	7	2.15	1	0.78	2	0.44
Wheezing	40	12.3	2	1.56	18	4.02

## DISCUSSION

In the hospital, individual patients are usually diagnosed to have COPD based on their clinical presentation and spirometry. In the field setting, in population-based surveys, spirometry may not be possible in many studies and data collection and interpretation is based on questionnaires. It is important to understand the strengths and limitations of the questionnaire used in the local population, before it is administered in the field. Different investigators have used different methods in estimating the prevalence of COPD. The most reliable studies have used a validated questionnaire along with spirometry, as spirometry helps to identify many early cases of COPD in the individuals who do not have any clinical symptoms and therefore will not be detected by the questionnaire alone. There are limitations with using spirometry alone as studies have shown that acute bronchodilator response has limited value in differentiating asthma from COPD.[21] Earlier studies in India have used questionnaires alone, questionnaires with PEF and only one study has used a validated questionnaire for the diagnosis of COPD.[22]

Respiratory symptoms are among the commonest clinical symptoms in the general population and may be due to various diseases. The classical definition of chronic bronchitis, which is a clinical definition with cough with or without sputum for at least three months in a year for at least two consecutive years, would be very simple to apply in field studies. However, various other chronic lower and upper respiratory diseases can give rise to similar symptoms. COPD has such varied presentations, many of which may not fit into the above simple definition. The population being studied also affects the diagnostic utility of the questionnaire. For example, selecting the subjects above 40 years of age for the study, rules out many nonCOPD cases, which may fit into the definition of chronic bronchitis, described above in younger age groups and thus, improves the diagnostic utility of a questionnaire to detect COPD. In the present study, the specificity of the questionnaire was probably enhanced by the definition used for COPD in both the stages of the study, which included not only the clinical symptoms, but also the presence of sufficient risk factors associated with COPD. The present study questionnaire showed an acceptable sensitivity and excellent specificity. A large study in Poland[23] on 1,10,355 subjects in the general population, showed that airflow limitation is noted in 23% of smokers aged >40 years and having smoked >10 pack years in the general population and 33% of these subjects did not have any respiratory symptoms. The lung health study[20] screened 73,000 smokers, aged between 35-60 years, and found that the airflow limitation in 30% subjects. These numbers for airflow limitation are far more than the prevalence of COPD in the general population. The study by Buffels,[24] which analyzed the usefulness of spirometry performed by general practitioners in early diagnosis of COPD, found that the number of newly diagnosed cases of COPD increased by 42% with spirometry compared to the diagnosis based on a questionnaire on signs and symptoms of COPD alone. These data clearly show that the addition of spirometry increases the number of COPD cases identified compared to questionnaire alone. In this light, the sensitivity of 62.5% for the validated questionnaire used in this study can be considered as excellent.

**Table 2:** Prevalence of COPD according to age, occupation, passive smoking (both at home and working place), cooking habits (females)

Variable	Classification	Prevalence			*P* value
		
		Number	No. of cases	%	
Age	40-49	174	7	4.02	<0.05
	50-59	118	7	5.93	
	60-69	87	4	3.39	
	70+	68	5	7.35	
Occupation	Unemployed/retired	0	0	0	<0.05
	Housewife (Females)	364	13	3.57	
	Unskilled worker	39	5	12.82	
	Skilled worker	10	3	30	
	Bussiness	3	1	33.33	
	Farmer	29	1	3.45	
	Government/private worker	2	0	0	
	Government/private supervisor	0	0	0	
	Government/private officer	0	0	0	
Exposure to smoke	Lpg	25	1	4	<0.05
	Kerosene	6	1	16.66	
	charcoal	0	0	0	
	Firewood	411	21	5.11	
	cowdung	0	0	0	
	Biogas	1	0	0	
	Lpg+firewood	4	0	0	
Passive smoking (At home)	Yes	246	14	5.69	>0.05
	No	201	9	4.47	
Passive smoking (At work)	Yes	36	7	19.44	<0.05
	No	411	16	3.89	

The information regarding prevalence of COPD in India is patchy at best and large parts of the country have not been covered. India is a large heterogeneous country with differing cultural and socioeconomic background and a recent review on COPD[22] prevalence, listed only 10 studies in the last 30 years. The studies were limited to the states of UP, Delhi, Punjab, Haryana, and Tamilnadu. Only one of these studies was conducted in the last decade. The lowest figures were observed by Thiruvengadum *et al.*,[7] in South India in Madras and reported prevalence rates of 1.9% in males and 1.2% in females. The highest figures reported in males were in Punjab, North India, by Joshi *et al.*[5] and reported a figure of 12.5%. In females, Radha *et al.*,[9] observed a prevalence of 4.6% in Delhi. A total of 3 out of 10 studies were conducted only in the rural population. The most recent of these studies by Jindal *et al.*,[3] was also the largest using proper epidemiological techniques, appropriate sample size, and sampling strategies and a validated questionnaire; and observed a prevalence of 5.0% in males and 3.2% in females. Our study reported a higher prevalence of 11.1% in males and 4.5% in females. This may be influenced by the fact that 71.9% males in this study were smokers and is higher than the national figures of 13.3 to 59.4% in men. In the recent study by Jindal *et al*., the average prevalence of smoking in men was 28.5%. More than 90% females in the study were exposed to biomass fuels for more than 20 years.

Smoking and biomass fuel exposures were significantly associated with COPD as were passive smoking and increasing age. These risk factors are similar to those identified in earlier studies. In India, smoking association is reported in 82.3% of male patients.[22] In our study, it was 96.4% of male patients and very few cases were observed in nonsmoking males. The population attributable risk of COPD in the OLIN (Obstructive Lung Disease in Northern Sweden) study[25] for smoking was 45%, less than the figures of 80–90% commonly quoted. The recent multicentric study by Jindal *et al.*,[22] reports a smoker: Nonsmoker ratio of 2.65:1. In our study, we observed a smoker: Nonsmoker ratio of 6.70:1. A dose response relationship was also observed in our study as described in earlier studies, where 9.6% smokers who smoked for less than 20 pack years had COPD (n = 125). The prevalence increased to 18% in those who smoked for more than 20 pack years (n = 200). In the OLIN study,[25] it was demonstrated that 50% smokers would develop COPD in their lifetime. This was a cohort study and raised important concerns about the risks associated with tobacco smoking. In our study, we found that 14.7% smokers developed COPD, which increased to 28.7% in the 70+ age group. Ours was a cross sectional study and it is important to perform longitudinal studies in India to understand the full implications of smoking.

Biomass fuels have been identified as one of the major risk factor for COPD in both developing and developed countries.[26,27] Up to 20% of COPD cases worldwide can be attributed to indoor air pollution from exposure to smoke from cooking and heating with biomass fuels in poorly ventilated dwellings. Smith *et al.*,[28] were the first to suggest that exposure to wood smoke could equal up to 20 pack years of active exposure to cigarette smoke. Dennis *et al.*,[29] showed that wood smoke exposure is associated with the development of COPD among females of low socio-economic status in Bogota (Colombia).

In a study in Spain,[27] one of the first studies of wood smoke exposure in a developed country, wood smoke exposure explained around 50% of all COPD cases. In a review of the health effects of Environmental Tobacco Smoke (ETS),[30] an increased risk of COPD was found. The excess risk related to ETS exposure was estimated to be from 60% to 400%. Dose-response relation was also noted.

In our study, most of the COPD cases in women were associated with exposure to biomass fuels. The prevalence of COPD in women using biomass fuels alone was 3.9% and increased to 4.8% when there was combined exposure to biomass fuels and passive smoking thus demonstrating an additive effect. Since 99.5% of women used biomass fuels, it was not possible to study the effects of passive smoking alone. The number of women using other fuels was very small and so no conclusions could be drawn about their association with COPD.

In a study in Morocco,[31] more than 70% of the smokers were aware of the respiratory and cardiovascular risk of tobacco smoking. In comparison, our population in the rural area had very little knowledge of the risks of tobacco smoking with only 24% aware that tobacco smoking is associated with respiratory disease and around 12% for cancer, less than 9% for heart disease, and less than 5% for other diseases. The proportion of male subjects who smoked in our study was very high at 71.7% and coupled with poor knowledge about the adverse effects of smoking are likely to have a significant impact on morbidity and mortality associated with smoking in this population. Larger studies are needed in the rural areas of the country to assess the knowledge regarding smoking and ill health and if found to be similar to our study demands an extensive education drive, if we are to control the epidemic of COPD in India in the coming decades. Most people who smoked did so for relief of stress and to keep company or for enjoyment. It is important to train the health staff of the primary health centers in the rural areas for counseling on the ill effects of smoking. Smoking cessation will have to be taken up on a large scale as a national control program.

In a study in Spain,[32] Fagerstorm questionnaire was used to assess characteristics in smokers with and without COPD. It was observed that smokers with COPD had higher mean Fagerstorm test scores, 4.77 (SD 2.45) compared to smokers without COPD, 3.15 (SD 2.38). The study concluded that smokers with COPD were more likely to have greater physical nicotine dependence than smokers who did not develop COPD. In our study, 54.5% smokers had mild to moderate nicotine dependence and 45.5% had severe nicotine dependence. The mean Fagerstorm scores in smokers with COPD were 6.29 (SD 2.76) compared to 5.20 (SD 2.73) in smokers without COPD. The prevalence of COPD among severe nicotine dependent smokers was 20.3% compared to 10.16% in mild to moderate nicotine dependence. Higher dependency scores were noted in our population than the subjects in Spain and we observed similar results identifying greater nicotine dependence as a significant risk factor associated with COPD.
